# DRD4 Gene Polymorphisms as a Risk Factor for Children with Attention Deficit Hyperactivity Disorder in Iranian Population

**DOI:** 10.1155/2017/2494537

**Published:** 2017-05-24

**Authors:** Seyed Mahmoud Tabatabaei, Shahrokh Amiri, Sara Faghfouri, Seyed Gholamreza Noorazar, Shahin AbdollahiFakhim, Ali Fakhari

**Affiliations:** ^1^Department of Physiology, Tabriz Branch, Islamic Azad University, Tabriz, Iran; ^2^Research Center of Psychiatry and Behavioral Sciences, Tabriz University of Medical Sciences, Tabriz, Iran; ^3^Department of Otolaryngology, Head and Neck Surgery, Tabriz University of Medical Sciences, Tabriz, Iran

## Abstract

**Background and Objective:**

Dopamine dysfunction is known to be associated with attention deficit hyperactivity disorder (ADHD). Dopamine D4 receptor gene (DRD4) is one of the important genes in this pathway. This study intended to investigate the variable number of tandem repeats (VNTR) in exon 3 of the DRD4 gene in Iranian children and adolescents.

**Materials and Methods:**

In this study, 130 children with ADHD, aged 6–14 years, and 130 healthy children, within the same age range, were enrolled. All children were selected from northwest of Iran which have Caucasian ethnic background and are of a Turkic ethnic group. VNTR polymorphisms of the DRD4 gene were evaluated by PCR using exon 3-specific primers followed by agarose gel electrophoresis.

**Findings:**

The Hardy-Weinberg principle and Chi-square test showed a significant difference in 4-repetition (4R) alleles between the ADHD (76.2%) and control (53.8%) groups (*p* = 0.004; *X*^2^ = 17.39; df = 5). The least percentage of repetition alleles in both groups was 2R.

**Conclusion:**

There is a significant correlation between the 4R alleles of DRD4 and ADHD in the northwest of Iran.

## 1. Introduction

ADHD is a common childhood psychiatric disorder that is associated with the symptoms of sustained hyperactivity, impulsiveness, and inattentiveness. The worldwide prevalence is 4–8% in school-aged children, which may persist during adolescence and adulthood in 50–80% of cases [1]. A comparable prevalence is reported from Iranian population and the rate is estimated to be 9.7% in children living in Tabriz city [2].

ADHD is a heterogeneous neurobehavioral disorder with multifactorial etiology, leading to neural pathway alternations [3]. The phenotype is quite wide and includes impaired social functioning and skill acquisition and decreased cognitive abilities. These increase the burden of undiagnosed and untreated ADHD with a significant impact on the career, life, and academic achievements [4, 5].

Diagnosing ADHD is currently based on clinical similarities, like other psychiatric conditions. The diverse phenotype results in diagnostic challenges [6, 7] that should be addressed by improvement of diagnostic methods. Toward this approach, pharmacological studies, animal models, and brain imaging all show the involvement of catecholamine pathways including neurotransmitters and related genes [8]. Dopamine and norepinephrine are involved in neurological functions, as well as attentiveness and awareness [8]. On the other hand, dopamine pathways are involved in motor control and in controlling the release of various hormones. These pathways and cell groups form a dopamine system which is neuromodulatory. The motor functions of dopamine are linked to a separate pathway, with cell bodies in the substantia nigra that manufacture and release dopamine into the dorsal striatum. DRD4 gene that is expressed in several brain areas has also been investigated in relation to ADHD [9, 10]. Linkage mapping shows the locus of this receptor gene on chromosome 11p15.5 [11]. This gene has several polymorphisms in its nucleotide sequence. The 48-base pair variable number tandem repeat (VNTR) in exon III of DRD4 polymorphism is the most studied polymorphism in association with ADHD. Biological molecular studies show that this region couples to G-protein, modulating cAMP production [12].

The length of exon III varies between 2 and 11 repeats of a similar 48-bp VNTR, presented by D4.2 to D4.11 [13]. Typically, these repetition alleles have two categories: short repeat length allele (2–4 repetitions) and long repeat length allele (5–8 repetitions) [14]. Meta-analysis shows that variants 2, 4, and 7 are far more common [15].

There are evidences supporting significant relationship between 7-repeat alleles of DRD4 exon III polymorphism and ADHD [16–19]. On the other hand, another group of studies have reported a weak correlation between DRD4 and ADHD [20, 21]. However, it is not well known if these negative results are due to the difference between groups and populations with different race, genetics, and heterogeneity, weakness in performing and interpreting statistical tests, or a real difference between different societies. As a result, this study aimed to investigate the relationship of DRD4 polymorphism with ADHD in Iranian population.

## 2. Materials and Methods

The present study was conducted in a child and adolescent psychiatry clinic of Tabriz University of Medical Sciences. The procedure was approved by the regional ethical committee and written informed consent was obtained from parents of participated children and adolescents.

## 3. Inclusion and Exclusion Criteria

The inclusion criteria were children and adolescent, aged 6–18 years, diagnosed with ADHD, based on clinical interviews conducted by a children and adolescent psychiatrist.

The exclusion criteria were concurrent psychiatric disorder, epilepsy, a serious medical condition or history of severe trauma, and any degree of intellectual disability. ADHD cases with comorbid ODD and/or CD were excluded.

## 4. Participants

This study was conducted in northwest of Iran. The populations living in these areas have Caucasian ethnic background and are of a Turkic ethnic group. Children diagnosed with ADHD (*n* = 130) in specialized psychiatric clinic were included by a child and adolescent psychiatrist. In this study, ADHD diagnosis was carried out through a semistructured diagnostic interview based on DSM-IV-IR criteria.

A group of children (*n* = 130), who were introduced for adenotonsillectomy procedure, within the same age range, were taken as the controls. These children were diagnosed to have no psychiatric condition by the same diagnostic procedure as children with ADHD. An informed written consent was obtained from all parents.

## 5. Instruments

### 5.1. Kiddie Schedule for Affective Disorders and Schizophrenia-Present and Lifetime Version (K-SADS-PL)

This is a semistructured diagnostic interview, designed based on criteria defined by the Diagnostic and Statistical Manual for Mental Disorders, 4th edition (DSM-IV). This tool was to identify ADHD and possible comorbid psychiatric conditions as well as choosing controls [22].

The diagnostic procedure was completed through interview with child and the parents. The test-retest and interrater reliability of the Persian version is reported to be 0.81 and 0.69 [22].

### 5.2. PCR-Variable Number of Tandem Repeats

In his process, 2 mL peripheral blood samples were prepared and genomic DNA was extracted from it, using QIAamp DNA kit according to the manufacturer's instruction. The extracted DNA was quality-checked by using a NanoDrop device within the range of 260–280 nm. The genotyping of DRD4 exon III was carried out by polymerase chain reaction with the following primer sequences:

  (Forward)* 5*′-*GGTCTGCGGTGGAGTCTG*-*3*′
  (Reverse)* 5*′-*GCGACTACGTGGTCTACT-3*′

Amplification by PCR was performed in 20 *μ*L reaction mixture containing 50 ng genomic DNA, using Ampliqon Master Mix (Denmark), 1 mL from each primer, and 0.2 *μ*LTaq DNA polymerase, according to temperature plan in Table 1. PCR products were observed in agar gel (2%) stained with ethidium bromide fluorescence under ultraviolet light. Digestion products were analyzed after agarose gel electrophoresis.

## 6. Statistical Analysis

Data from counting the gene products in each sample was fed to SPSS21. Data are expressed as number and percentage, and the obtained mean values are investigated, using the Chi-square test. In this study, *p* < 0.05 was considered significant.

## 7. Results

One hundred and thirty children diagnosed with ADHD and 130 children with no psychiatric diagnosis were enrolled. Mean age of children in the two groups was not significantly different. However, there were more males in the ADHD group (83% in the ADHD versus 61% control group). The role of a first-degree family relationship was present in many samples. Descendants of a cousin marriage accounted for the majority of children with ADHD (Table 2). As the results show, the parents of ADHD children had a significantly higher proportion of first-degree relatives. Perhaps its cause is that there was a different pattern than the multifactorial pattern for this disorder. The study of pedigrees shows single-gene inheritance pattern; that is why the frequency of children with consanguineous marriage (especially the first cousins) was more than the control group.

Table 3 shows the frequency of VNTR DRD4 in the ADHD and control groups. The Hardy-Weinberg principle and Chi-square test showed a significant difference in 4R alleles between the ADHD (76.2%) and control (53.8%) groups (*p* = 0.004; *X*^2^ = 17.39; df = 5). There was no significant between-groups difference in other repetitions. The 2R genotype had the lowest prevalence.

## 8. Discussion

ADHD is a prevalent neurobehavioral disorder starting during childhood [23]. According to several meta-analysis studies, genes involved in dopaminergic pathway, specifically DRD4, have an important role in pathogenesis of ADHD [16–18]. There is no available study on the relationship of this topic in Iranian population and this study was conducted to use repetitions of this gene as a biomarker for facilitating the diagnosis of this disorder in children.

In the present study, the DRD4 gene was observed in all Iranian participants (ADHD and controls) within the range of 2R to 7R alleles. The dominant alleles in our study were 4R, 5R, and 6R, among which the 4R allele had the highest prevalence. The between-groups difference was significant only in 4R allele. Results of the present study are consistent with those of Bidwell et al. [19]. Cheuk et al. showed that the 4R allele had the highest frequency (84%) among their research population; in addition, this genotype was correlated with presence of ADHD [24].

Though these studies were in line with the present study considering 4R as an indicator of ADHD, studies on other populations, specifically White Europeans and Americans showed a greater role of 7R alleles in the development of ADHD [25]. In contrast, our study did not show any significant relationship between 7R alleles and ADHD.

Another meta-analysis study showed a significant difference in the relationship of ADHD and DRD4 7R in people of European-Caucasian and South American descent versus people of Middle Eastern Populations [26]. Leung et al. reported a very low prevalence of 7R alleles among Asians, whereas 4R had higher prevalence among them [27]. This report is consistent with the findings of our study.

According to demographic and ethnicity studies, it can be concluded that phylogenetics can be studied by investigating the polymorphism of genes in specific diseases. As it is seen, 4R is more prevalent in Asians than White Caucasians and Europeans [14]. High prevalence of 4R reported by Shahin et al. in Egypt [25], as well as our study confirms this finding.

However, there are studies that refuse the relationship of 7R alleles with ADHD. Carrasco et al. reports a negative relationship between 7R alleles and ADHD in a study conducted on a Chilean population [28]. Brookes et al. in a study in Taiwan reported no relationship between DRD4 biomarkers and ADHD [29].

The most obvious explanation for dissimilarity of reported results is differences of the study samples. However diagnostic procedure and methodological differences should also be accounted. The importance of a conclusion is not restricted to facilitating the diagnostic procedure but also might lead to preparing a precise treatment plan for each individual. Functional differences in the DRD4 intracellular signaling system have been studied for the 48-bp repeat alleles, and studies show that the 7R allele may be less sensitive to endogenous dopamine and mediates a blunted response to dopamine [12]. If results of the present study replicate in further studies, they might indicate good therapeutic response and better prognosis of ADHD in northwest of Iran.

It can be stated that the 4R allele carriers, which appear frequently in the ADHD patients in the northwest of Iran, may have a better prognosis than the carrier 7R, which in other populations have been linked to ADHD. In other words, the 4R allele repeats in DRD4 gene can be considered as a prognostic diagnosis in ADHD disorder in this area of Iran.

## 9. Conclusion

In contrast to the results of studies conducted on White Europeans and Americans, in which 7R was the genetic indicator of ADHD, our study showed 4R as the variant of this disorder in northwest of Iran.

## Figures and Tables

**Table 1:** Temperature plan used for proliferation of DRD4 exon III.

Cycle number	Stage	Temperature	Duration
One	First denaturation	95°C	5 min

Thirty-five	Denaturation	93°C	20 sec
	Annealing	55°C	20 sec
	Extension	72°C	30 sec

One	Final extension	72°C	7 min

**Table 2:** Demographics of the study sample.

Index	ADHD (*n* = 130)	Control (*n* = 130)	*p* value
Age (year)	6.883 ± 2.27	6.98 ± 2.50	0.3
Gender (boy/girl)	108/22	84/46	<0.001
Percentage of ADHD boys	83	61	0.04
Children of first-degree family marriage (%)	56	4	<0.001

**Table 3:** Frequency of variable number of tandem repeats (VNTR) in exon 3 of the DRD4 gene in Iranian children.

Number of repeats	ADHD group		Control group		*p* value
	Number	Percentage	Number	Percentage	
2R	3	2.3	9	6.9	>0.05
3R	4	3.1	16	12.3	>0.05
4R	99	76.2	70	53.8	0.004
5R	9	6.9	12	9.2	>0.05
6R	8	6.2	11	8.5	>0.05
7R	7	5.4	12	9.2	>0.05
